# Decoding of Turning Intention during Walking Based on EEG Biomarkers

**DOI:** 10.3390/bios12080555

**Published:** 2022-07-22

**Authors:** Vicente Quiles, Laura Ferrero, Eduardo Iáñez, Mario Ortiz, José M. Azorín

**Affiliations:** 1 Brain-Machine Interface System Lab, Miguel Hernández University of Elche, 03202 Elche, Spain; lferrero@umh.es (L.F.); eianez@umh.es (E.I.); mortiz@umh.es (M.O.); jm.azorin@umh.es (J.M.A.); 2 Instituto de Investigación en Ingeniería de Elche-I3E, Miguel Hernández University of Elche, 03202 Elche, Spain

**Keywords:** intention turn direction, EEG, BMI, ASR, *H* ^∞^, real time

## Abstract

In the EEG literature, there is a lack of asynchronous intention models that realistically propose interfaces for applications that must operate in real time. In this work, a novel BMI approach to detect in real time the intention to turn is proposed. For this purpose, an offline, pseudo-online and online analysis is presented to validate the EEG as a biomarker for the intention to turn. This article presents a methodology for the creation of a BMI that could differentiate two classes: monotonous walk and intention to turn. A comparison of some of the most popular algorithms in the literature is conducted. To filter the signal, two relevant algorithms are used: $H^{\infty }$ filter and ASR. For processing and classification, the mean of the covariance matrices in the Riemannian space was calculated and then, with various classifiers of different types, the distance of the test samples to each class in the Riemannian space was estimated. This dispenses with power-based models and the necessary baseline correction, which is a problem in realistic scenarios. In the cross-validation for a generic selection (valid for any subject) and a personalized one, the results were, on average, 66.2% and 69.6% with the best filter $H^{\infty }$. For the pseudo-online, the custom configuration for each subject was an average of 40.2% TP and 9.3 FP/min; the best subject obtained 43.9% TP and 2.9 FP/min. In the final validation test, this subject obtained 2.5 FP/min and an accuracy rate of 71.43%, and the turn anticipation was 0.21 s on average.

## 1. Introduction

In the field of rehabilitation, year after year, efforts are increased to create therapies that actively involve the subject in the process [1]. In addition, it is about standardizing these therapies based on biomarkers that allow us to indicate in some way the level of involvement of the subject [2] and the performance of the therapy. Non-invasive brain activity recording technology has proven to be effective in this field [3], proposing a path between the subject’s intention and motor action [4]. This connection between cerebral activity and machine action is called brain machine interfaces (BMI). Depending on the potential that is used, the BMI could have a more rehabilitative or more assistive role. However, the two purposes can be combined [5].

BMIs have been demonstrated as being able to decode brain motor patterns related to the upper and lower limbs in a large number of users with high accuracy, for both motor imagination (MI) [6] and intention [7]. BMIs can also decode up to four motor tasks with deep-learning techniques in which non-specific models are applied [8]. However, making the control depending on the motor task can be cognitively very demanding. Models of relaxation vs. motor imagination have shown promising results [9], but the performance still has room for improvement. Both paradigms have demonstrated their ability to operate in real time for upper and lower limbs: MI up [10], MI low [9]), intention up [11] and intention low [12]. Intention and MI paradigms have a different mental workload, which could be useful depending on the application. In rehabilitation, assuring that the subject is actively engaged in the MI could be positive to enhance neuroplasticity mechanisms [9]. However, in the assistive environment, MI can have high cognitive demands, unlike intention, which is the way people make decisions (spontaneously) and seems a natural paradigm for device control.

For the voluntary intention paradigms, two potentials are related to the preparation of the task: movement-related cortical potential (MRCP) [13] and event-related desynchronization and synchronization (ERD/ERS) [14]. The first paradigm is associated with low frequency changes (0.05–3 Hz) and the second with changes of power in alpha (8–12 Hz) and beta (13–30 Hz) bands. MRCP provides timing information about movement stages and planning execution, but it is very unstable against noise, and it is hard to detect it in single trial analysis [13]. On the other hand, ERD/ERS does not provide precise timing information about different stages of movement planning, preparation and execution, but ERD/ERS has been shown to be detectable from single trial EEG [15], and it is affected by noise in a minor way in comparison to MRCP [16].

Regarding the works in the bibliography, four types of analysis are usually carried out. Due to their difficulty from least to greatest, the first ones are the most abundant in the bibliography: offline studies with specific windows, pseudo-online analysis with a window-by-window sweep of the entire signal, real-time analysis in open-loop control without feedback, and real-time closed-loop analysis with feedback.

For the intention paradigm, several aspects have to be taken into account:

- Many studies present pseudo-online analysis with filtering steps that cannot be applied in real time or are unrealistic for their application.
- Creation of the model with instants only before and after the real movement or before the real movement. Regardless of how the model is created, the verification paradigm may be different. For example, in [15,17], an intention prediction verification model is applied, but the model is created with before and after instants.
- Level of cognition, totally spontaneous, planned, conscious or evoked (the subject is forced to turn when seeing a mark).

From offline studies with turn direction, the work carried out in [18] applied a large concatenated feature vector and trained the model with a k-nearest neighbors classifier. These studies obtained an offline accuracy of over 90.0% in most of the subjects. However, the methodologies are not suitable for pseudo-online.

Regarding the works that present pseudo-online analysis, the most abundant are those that propose start-walk and stop-walk paradigms. In [19], a model approach was made with a signal before and after the change and a processing based on the Stockwell transform. The evaluation was not predictive, and it used a four-second detection window for pseudo-online start and stop detection, which employed data from two seconds before the movement to two seconds after the movement. They achieved an average of 80.5 ± 14.6% True Positive (TP) with 4.43 ± 3.67 FP/min in start–walk detection and 84.1 ± 14.6% TP with 4.7 ± 5.3 FP/min in stop–walk detection.

However, in [15], a model with a signal before and after was created with the pseudo-online evaluation being predictive. Four data frames of each trial were taken as the baseline to calculate the ERD features. Nevertheless, the preprocessing methodology cannot be applied online, as artifact subspace reconstruction (ASR) and the ICA methodology were applied. The results for start–walk were 78.3 ± 8.6% and 6.5 ± 0.8 FP/min, and for the stop–walk paradigm 81.4 ± 7.2% and 9.2 ± 1.8 FP/min. This baseline was reported as a gap for real-time operations [15].

The literature shows two open-loop turn models with an exoskeleton, using the MRCP paradigm and evoked potentials. In [20], an algorithm, $H^{\infty }$ filter, was applied to mitigate ocular artifacts, achieving an accuracy of 98.17% for the turns with an impaired patient. In [21], ASR was applied as a preprocessing technique, to distinguish between four tasks, including turns to both sides. Two subjects participated in the study with different accuracies: able bodied (right 84.5% and left 78.5%) and impaired (right 72.1% and turn left 66.5%).

Regarding speed paradigms. In [22], the model was trained with a planned conscious model. The pseudo online model performed better with a window, including before and after data than with just the previous one. In [12], an only-before-data model was used both for training and testing. The results were 42.0% TP and a FP/min of 9.0 for the pseudo-online analysis. In [17], a model was trained with data before and after the motion. However, only data after motion were used in the evaluation. The preprocessing consisted of principal component analysis (PCA) + Laplacian spatial filter and the processing of a wavelet synchro squeezed transform with baseline correction. Results for a subject achieved 81.9 ± 7.4% with 7.7 ± 0.8 FP/min. In addition, a real-time test of 12 repetitions was completed with a 75% TP ratio and 1.5 FP/min. However, the EEG classifier was combined with an inertial sensor classifier working together with the predictive BMI.

When a BMI works in real time, the most crucial metric is FP/min. A high FP/min means that the interface will fail against the subject’s will too many times. If this happens in a closed-loop scenario, the BMI will force the changing of state. However, not many studies have addressed why and how often these FPs happen throughout the trials. It is important to analyze if the FPs are concentrated in a short period of time or if they are dispersed among the trials. In the case that the analysis is taken without feedback (open-loop control), the session can become monotonous. This can make the subject lose their focus, lowering the performance, just because the degree of alertness and vigilance are not proper [23]. Therefore, there could be trials with too many FPs and some without them [24], which may indicate that in an online test, the metrics could be more favorable. The analysis of the distribution of the FPs during the trials is one of the main points addressed in this work.

Several of these mentioned works have approached the paradigm of intention with different methodologies and purposes. There are not enough works that address an asynchronous intention model with pre-motion information, and its validation in real time with leading pre-processing (such as ASR [25] or $H^{\infty }$ [26]) and processing tools (such as Riemannian manifold [27] or common spatial patterns (CSP) [9]) in the literature. Therefore, the objective of this study was to use the EEG as a biomarker of the intention to turn. This indicator was used in real-time detection and in the future may be used to send commands to an assistive device. To achieve this objective, the present work is organized with the following scheme:

- Development of an algorithm for the proper detection of the moment of direction change through inertial measurement units (IMUs). This new algorithm provides accurate information of the turn, which allows an adequate segmentation of data for a proper intention model creation (only before information) and a correct metric evaluation of the intention to change direction.
- Implementation of state-of-the-art preprocessing techniques to mitigate artifacts. ASR and $H^{\infty }$ are compared in the paper.
- Development of robust methods for the distinguishing between monotonous walk (several seconds before change intention) and the intention to change direction (seconds before actual change of the walking direction).
- Offline analysis of brain patterns using the covariance matrices at different frequencies with a Riemannian manifold approach. The validation of these offline markers is tested online for one of the subjects to verify their consistency.

## 2. Materials and Methods

### 2.1. Equipment

**Figure 1:**
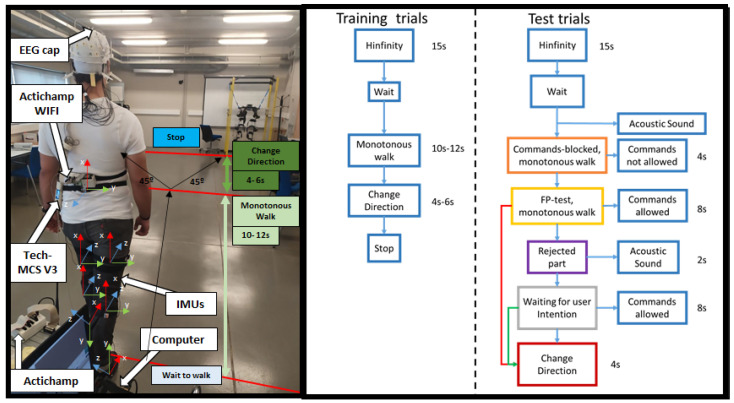
In the image on the left, the subject with the equipment on the day of the test. With this setup, the training and test were performed. The training and test protocol is schematized in the figure on the right.

For the EEG data recording, a 32-electrode actiCHamp bundle (Brain Products GmbH, Germany) was used (Figure 1). Signals were transmitted wirelessly by the Move transmitter to the actiCHamp. The electrodes registered EEG activity through 27 non-invasive active electrodes following the international 10-10 system distribution (F3, FZ, FC1, FCZ, C1, CZ, CP1, CPZ, FC5, FC3, C5, C3, CP5, CP3, P3, PZ, F4, FC2, FC4, FC6, C2, C4, CP2, CP4, C6, CP6, and P4). One of the electrodes was placed on the right ear lobe for reference, and an additional electrode acted as ground on the left ear lobe. The other four electrodes were placed to record electrooculography (EOG) activity in bipolar configuration (HR, HL, VU, and VD). A high-pass filter at 0.1 Hz and a notch filter at 50 Hz to remove the network noise were applied by hardware. Signals were registered at 500 Hz. A medical rack was placed on the head to mitigate the electrode and wire oscillation due to motion. The 32-electrode signals were visualized in real time by the technical assistant in the pycorder actiCHamp software during the trials.

For the change direction detection, seven IMUs (Tech MCS V3, Technaid, Spain) were placed for registering the walking parameters at 50 Hz sampling (Figure 1 left): one at the back and for the remaining six, three on one leg and three on the other placed on the gluteus, shin and foot.

EEG and IMU signals were recorded at their sampling frequencies and synchronized in Matlab by a custom software.

### 2.2. Register Experimental Procedure

For the experiment, the subject trained a model of brain activity. The experiment was designed to try to discriminate among two classes: monotonous walk and intention to turn. To characterize these mental states, the subject was instructed to leave their mind blank out. The subject should turn at their will trying not to anticipate the changing too much to assure an spontaneous change of direction.

At the beginning of the session, 60 s of signal were recorded (for the ASR baseline) while the subject was standing and as much relaxed as possible (basal state). Subsequently, the subject performed 13 trials of the designed protocol for the recording of spontaneous EEG related to direction change. In each of these trials, the subject started standing. During the initial 15 s, the activity was recorded in basal state for the $H^{\infty }$ convergence. Once this period was over, the subject started walking until they decided to turn left or right indifferently approximately at a 45 degree angle. The subject was instructed to turn abruptly (turn with the whole body, instead of starting with one leg or with the head to facilitate detection with the back IMU). The turn was made asynchronous.This was done within the trial, without stopping the recording eight times (Figure 1 Right Training trials), so each trial consisted of eight turn event repetitions.

Nevertheless, one turn event repetition could be discarded for the creation of the model if an anomaly was observed. Anomalies detected could consist of forgetting to turn, making a strange gesture, coughing, biting or chewing, swallowing, or turning very soon and not allowing enough normal walking time period (details in Section 2.4.2).

The first two trials were discarded, as the subjects were getting used to the protocol. Subjects performed around 12 trials, as 10 was the number of trials employed for the analysis. The last trial (number 13) was carried out with the aim of taking a similar number of repetitions through the subjects in case more repetitions were discarded. This last repetition was only added if the number of discards exceeded 10 repetitions.

### 2.3. Subjects

Eight healthy subjects, with an average age of 25.4 ± 3.5 years (four female and four males) participated in the study. The subjects reported no diseases and they participated voluntarily in the study by giving their informed consent according to the Helsinki declaration approved by the Ethics Committee of the Responsible Research Office of Miguel Hernandez University of Elche (Spain).

After analyzing the signal, one subject was selected to validate online the proposed model in real time.

### 2.4. Offline Validation of the Turn with the Imus

#### 2.4.1. Algorithm to Detect the Turn

Of the seven sensors recorded, only the back IMU was used for gyro analysis. The choice in this paper of a single IMU rather than a combination of a group of IMUs was due to the fact that turn strategies are usually top–down, and there may be a time lag in the accurate detection of the turn moment. Thus, depending on the position and measurement (angle or angular velocity), the noise can affect differently. In [28], the back position (upper or down) was reported as the most effective one, being that the angle measurement is the most accurate way to detect the turn. Therefore, to standardize the turning methodology and assure that the body trigger was the same for all the subjects in this research, they were asked to turn with the trunk.

From the matrix of leading cosines, the *Xz* and *Yz* values were used and the angle in the zenith plane was calculated, according to the following equation:

(1) $$Angle=tan_{}^{-1}\left(\frac{Xz}{Yz}\right)\frac{180}{\pi }$$

In order for the algorithm to be accurate and calculate the exact point where the major angle change occurred, the noise was reduced by using a *mslowess* filter in matlab with ‘Order’, 0, ‘Kernel’ type ‘Gaussian’ and ‘Span’ smooth window value of 0.02.

The inflection points were calculated from the filtered signal. First, the double derivative of the signal was calculated, and a threshold was selected based on the maximum value that produced the inflection of the turn. For obtaining the threshold, it was iteratively evaluated which inflection values exceeded the average of the inflection values by a factor. This factor started at six; if no value fulfilled the condition, the factor was decreased by one, until it reached one. For the values above the mean, the first one was temporarily chosen, and this was selected as the change point.

**Figure 2:**
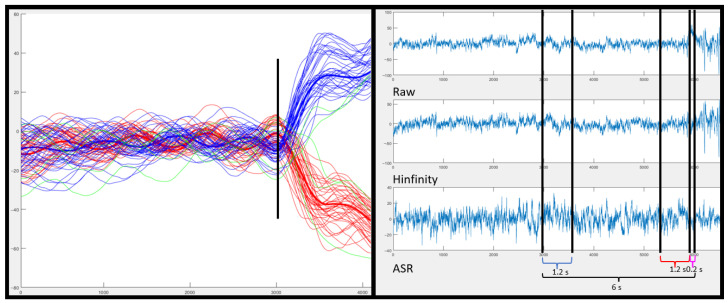
*On the left*: Image of the processed IMU signal. In black, the moment classified as turn. In blue highlighted, the average number of turns on the left and in red highlighted, the average number of turns on the right. In green, the turns that did not meet the criteria assigned to the turn type (left or right) assigned by the algorithm. *On the right*: the EEG signal of the third turn for the Fz channel of subject S2. Labeled with respect to the turning point marked on the IMUs. For the segmented class in blue monotonous walk and for the intention turn class in red. According to the raw EEG signal (first image), the signal filtered with $H^{\infty }$ (second image) and the signal filtered with ASR (third image).

Repetitions were labeled according to its directions as left or right. For this, the mean of the signal before the change and the mean of the signal after the change were averaged. If the value of the mean after the change is positive, it is considered a right turn; otherwise, it is considered a left turn (see Figure 2).

EEG data labeling was based on the actual point at which the turn was detected by the IMU. The monotonous walk class was considered a 1.2 s window, six seconds before the turn, previous to the change and change direction intention classes (Figure 2). The temporal specification of these windows is detailed in Section 2.5.1.

#### 2.4.2. Discards and Validation

Within the protocol-related discards, IMUs were used to discard those repetitions in which the subject turned too fast. The criterion chosen was that the distance between the third step (labeled as in [29]) and the turn should be less than 6 s. In addition, a turn could be rejected if it was labeled incorrectly. A discard algorithm was designed to flag those turns that disagree with a criteria based on the means of the left and right repetitions. Each repetition was correlated with the mean and considered erroneous (in green Figure 2) if the correlation index was lower than 0.9, or the angle increment differed by an angle lower than 25 degrees from the mean signal.

Because the algorithm can discard more repetitions than it should, for the creation of the offline-cross validation model in the previous analysis of the EEG signal (before the online test) only those that were visually detected as erroneous were discarded, and the discard algorithm was only used as an aid. After validation (Section 3.1), it was used in the real-time test (Section 2.7).

Due to the fact that the algorithm can discard more repetitions than it should, for the creation of the Offline-cross validation model in the previous analysis of the EEG signal (before performing the online test), only those that were visually detected as erroneous were discarded. In this section, the discard algorithm is only used as a tool for helping the visual discard. After being validated (Section 3.1), it was used in the online test (Section 2.7), where time was more critical and in a hurry, the visual inspection could not be performed correctly.

### 2.5. Offline and Pseudo-Online Validation of the EEG Intention

The aim of this work is to use the EEG as a biomarker of subject intention and use it on its own, independent of the signals from the IMUs. The acceleration signal is used to generate the predictive EEG model from the IMUs as a marker of the actual turn execution.

#### 2.5.1. Offline-Cross Validation

The turns were segmented according to whether they were leftward or rightward. However, intention patterns of monotonous walk vs. turn were classified for both left and right turns in the EEG analysis. To find out which configuration maximized the classification results, a leave-one-out cross-validation was computed. Different artifact filters, frequency bands and classifiers were tested.

Before segmenting the signal, two types of filters were applied to the entire recording separately:

- $H^{\infty }$ which mitigates ocular artifacts and signal drift. It is applied epoch by epoch by state variables (Figure 2 Right).
- ASR algorithm needs 60 artifact-free pre-signal and mitigates possible EEG artifacts of the online signal each 0.2 s.

To apply both algorithms, frequency filters by state variables (order 2) were employed to isolate different bands: 8–14 Hz, 15–22 Hz, 23–30 Hz, 31–40 Hz and 8–40 Hz. The selection of these bands was based on the basic connections of the nature of the EEG [30] and previous works in which the intention was characterized [14,22].

To characterize the time windows of both the monotonous walk and turn intent classes, the previously calculated turning point was used as a reference point 0 s. The monotonous walk class was defined in a period of time between −6 s and −4.8 s before the actual event. The change intention class was defined within −1.4 s and −0.2 s before the actual turn. Later, the covariance matrix features of all electrodes were extracted according to the “scm” method for each artifact filter condition and band in all the segmented windows.

From the 10 trials considered valid, the first eight trials were used in the validation (one for testing and seven for model). The last two trials were left for the pseudo-online testing explained in Section 2.5.2.

The Riemannian paradigm was used for modeling. This paradigm was applied for four different classifier types: a support vector machine (SVM) classifier [31], a minimum distance to mean (MDM) classifier, an MDM classifier with filter and an linear discriminant analysis (LDA) [27]. The evaluation metrics used for the classifiers were accuracy (*Acc*) and balance (*b*) between classes. The randomness value was used to filter out those Acc that were below the randomness threshold [32] and those *b* that were above 10 (this value was considered as a good tolerance between classes difference for a balance classification prediction).

(2) $$Acc=\frac{\mathrm{Number}\;\mathrm{of}\;\mathrm{true}\;\mathrm{event}\;\mathrm{detection}}{\mathrm{Number}\;\mathrm{of}\;\mathrm{total}\;\mathrm{event}\;\mathrm{detection}}$$

(3) $$b=abs(\frac{AccClassChange-AccTotal}{AccClassChange})$$

(4) $$RandomnessLevel=0.5+1.96\times \sqrt{\frac{0.5_{}^{2}}{NumberofValidRepetition+4}}$$

The values that passed this cut-off were averaged depending on the classifier, according to the artifact filter type and band filter. From this analysis, a generic configuration valid for all subjects will be chosen and will also be compared with a personalized one, that is, the best band and classifier configuration for each subject. The relevance of these two configurations in real-time testing will be discussed later.

In addition, several alternatives were tested in the best configuration of these two to discuss how they can influence, for example, creating an intention prediction model only with right turns or only with left turns and if there is any difference with the current model that includes right and left turns. In addition, the contribution of the different brain areas to our classification model were analyzed.

##### Offline-Cross Validation Model Type

According to the criteria discussed in Section 2.4.1, the repetitions were grouped according to whether the turn occurred in the left or right direction, and a cross validation was carried out with fewer testing trials than in the left and right model.

##### Offline-Cross Validation Electrode Selection

From the personalized configuration, it was also studied which channels have a greater difference between the classes of monotonous walk and change intention from the covariance matrix. To calculate the electrodes that maximized the difference between the covariance matrices of both classes, the mean of both covariance matrices were calculated. For the monotonous walk class *MeanClassMonotonousWalk* and for the change class *MeanClassChange*, the formula used was the following:

(5) V=abs(MeanClassChange−MeanClassMonotonousWalk)

From the diagonal array, the highest pairs of electrodes were taken and added to the list. This list was ordered from highest to lowest, and the first 15, 10 and 5 highest were taken.

#### 2.5.2. Pseudo-Online Analysis

After detecting the best combination of features and electrode configurations for the three types of classification models, the pseudo-online analysis was performed in order to check them in a simulated real-time scenario.

No cross validation was used in the pseudo-online analysis in order to simulate the real-time conditions. This way, the model was created using the first eight trials with the explained offline procedure and tested with the last two trials of each session with the following procedure.

Trials were analyzed epoch by epoch, with a size of 600 samples (1.2 s), shifting them every 100 samples (0.2 s), producing an effective 1.0 s overlap between epochs. The preprocessing was performed in the same way as in the offline model using the state variable filters. However, the whole signal was processed in the pseudo-online analysis extracting the features based on the generic and personalized selection.

**Figure 3:**
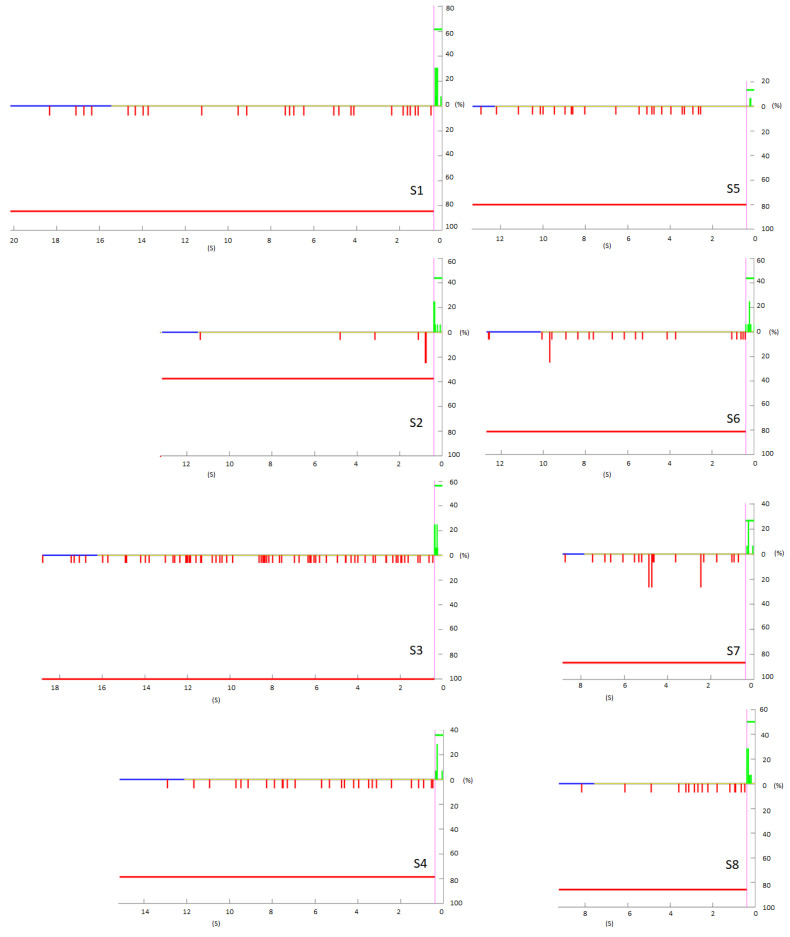
Pseudo-online with 16 repetitions. Horizontal line at 0, in yellow the mean of the first moment in which the PFs are evaluated. In blue, the temporary maximum where it is evaluated in yellow the mean of the time repetitions duration. The FPs are shown in red as they happened temporarily with respect to the change in the X axis, the value of Y is based on the percentage of times that they appeared at that moment. The red horizontal line is the average number of trials in which an FP appeared. Analogously, the TPs are shown in green and the average PT horizontally.

Meanwhile, the classification of each window was only evaluated in the interval between points marked as the beginning of walking (the third step) and the change labeled (see Figure 3). Regarding the command decision, a TP was computed if five consecutive epochs were identified as a turn change class and the detection was detected at most 0.4 s before the actual IMUs detection of the change; otherwise, it was considered a FP. Taking into account the epoch size and shifting, the time window in which a TP can be considered valid before the actual change could contain information from −2.40 s to 0 s.

The number of successful changes, the *TP* rate of the test, could be defined as

(6) $$TP=\frac{\mathrm{Number}\;\mathrm{of}\;\mathrm{true}\;\mathrm{event}\;\mathrm{detections}}{\mathrm{Number}\;\mathrm{of}\;\mathrm{true}\;\mathrm{events}}$$

If one of the epochs was detected within the change range, a true event was computed, and the signal processing was stopped until the start of the next repetition. As a realistic way to compute FP, once one was detected, the next one could not be computed until at least two seconds passed. This helps to simulate that in a real-time scenario, as the command is sent (to perform a feedback action), it is necessary to wait a while until the next command can be sent again. As the normal walking time period has a duration of 8–10 s, the FP evaluation can be expressed as

(7) $$FP/min=\frac{\mathrm{Number}\;\mathrm{of}\;\mathrm{false}\;\mathrm{activations}}{\mathrm{Walking}\;\mathrm{time}\;\mathrm{windows}\;\mathrm{in}\;\mathrm{minutes}}$$

In addition, the FPRatio was calculated, which indicates the percentage of repetitions in which there was an FP. The TPnoFP indicates the percentage of repetitions in which there was no FP and if there was TP.

### 2.6. Online Experimental Procedure

With the offline register protocol, the intention patterns were analyzed and studied in eight subjects to finally propose an online evaluation (Figure 1 Right Test trials): On the day of such an evaluation, first a model was again created with the day’s activity in an analogous way as explained above (Section 2.2). This model was created with fewer trials, since the subject is already trained in this task. The model was then generated with the selected features and filters; the EEG analysis methodologies are discussed in Section 2.5.1. Finally, the effectiveness of real-time BMI prediction was evaluated:

In each online trial, at the beginning, the subject waited and relaxed 15 s for the $H^{\infty }$ to converge, and after this period, the subject walked four seconds (no FP were allowed) + eight seconds (FP were allowed) without the intention to turn to evaluate FP. Subsequently, after the instructional cue, the subject decided in a time interval of three to five seconds when they wanted to turn. If the turn happened before the detected turn, it was counted as a TP turn. The details of the validation are discussed in Section 2.7.

### 2.7. Online Validation of the EEG Intention

An online test was performed with one of the subjects who already had experience with the protocol. The training was carried out in the same way than the offline model. To create the model, it was necessary to choose the most reliable artifact filtering technique to apply, the frequency band which provides the best segmentation, and the classifier which performs with a higher accuracy. This choosing can be based on two types of approach after analyzing the offline results. First option consists of a general configuration, which would be valid for most of the subjects and that does not require a high temporary expense. The second option looks for maximizing the success, personalizing the model for each of the subjects. The second approach, as it must be done between training and testing, can take a long time. In this study, the explained configuration took about 15 min, so it was chosen as the valid option.

For signal analysis, the features were extracted from windows of size 1.2 s, and the window moves every 0.2 s in real time. However, in the pseudo-online analysis, it was considered a mode of the window of five. This was adapted to the online test. For the specific test, a mode of four was used. For this adaptation, in the first two repetitions, it was analyzed which mode was the most suitable to maximize success in the 2nd phase.

Once the model was created following the steps below: algorithm IMUs detection, algorithm discard IMUs and offline-cross validation for best configuration selection. The protocol consists of two phases: As explained above, the subject started walking after the 15 s of convergence of the $H^{\infty }$. In this 1st phase, no FP should be detected, failing if one is detected. A FP is computed if the interface detected the class intention to turn in the verification metric instead of the class monotonous walk.

If the 1st phase did not compute FPs, the second phase was considered TP if the turn intention was detected before the IMUs algorithm detects the change (see Section 2.4.1). A FP at the 2nd phase was considered if the interface detected a change after the IMUs detection or 0.6 s before the turn occurred in the period of monotonous walking, the window interval for the pseudo-online test was 0.4 but due to the change of mode (from five to four), these were readjusted.

The evaluation was computed in real time, but the feedback to the subject was given by the technical assistant, indicating the subject to turn (closed-loop) if the interface failed in the first phase. If during the evaluation phase, the BMI detected a TP, the technical was able to visualize it and gave feedback on the performance after the voluntary turn was done (open-loop), as the difference in time was so close such that it prevented the real-time feedback by voice command. Nevertheless, a verification of the TP was performed after the trial.

## 3. Results

### 3.1. Offline Validation of the Turn with Imus

Table 1 shows the number of trials discarded according to the different final criteria for the offline-cross validation analysis. The last column indicates the actual number of valid repetitions in the 8 of the 10 trials selected for offline cross validation.

The discard algorithm for the IMUs validation was designed first to identify the repetitions wrong labeled by the algorithm. With this information, the repetitions were visualized and discarded if considered erroneous. In addition, those that failed by protocol or in which there was not enough time between classes were discarded. The number of repetitions discarded in each case is specified in the Table 1.

Second, the discard algorithm was used to speed up the discarding of repetitions process in the test in real time. For this, it was first validated with the records of the eight subjects, comparing the visually discarded repetitions with those that were discarded by the algorithm. In this way, in Table 2, three rows are shown belonging to the number of repetitions that the algorithm discarded and are also visually discarded (both), the number of repetitions that were discarded by the algorithm and not by visual inspection (only algorithm) and the number of repetitions discarded by visual inspection and not by the algorithm (only visual inspection).

The discard algorithm agreed with the visual inspection in all cases, except S1 where it failed in one prediction. The algorithm was predicted as wrong in all cases more than the visual selection, except in the case of S3. On average, it predicted four more errors than the visual inspection.

### 3.2. Offline and Pseudo-Online Validation of the Turn Intention with EEG

For the design of the BMI, first the results of the offline validation are presented. Then, the results of these configurations are shown in a pseudo-online evaluation. Finally, with the selected configurations, the results of the online test are presented.

#### 3.2.1. Offline-Cross Validation

Two different types of filtering ($H^{\infty }$ or ASR) were studied as preprocessing filters. Furthermore, four types of Riemannian classifiers (SVM, MDM, MDMfilter and LDA) were chosen to classify the mental patterns of intention to turn and monotonous walk from different EEG frequency bands.

To evaluate the best configurations, a statistical analysis was applied in R. The whole *Acc* data from the cross validations were used as data in the analysis. To see the differences in *Acc* between subjects, regardless of the configuration, a Shapiro–Wilk test was applied to assess the normality assumption and homoscedasticity of ANOVA. Differences between subjects were significant at a *p*-value < 0.01. The normality study was also applied as an assumption of repeated-measures ANOVA to see the differences between $H^{\infty }$ and ASR filters. Differences between filters were not significant at a *p*-value < 0.01.

Another repeated-measures ANOVA was applied to look for differences between classifiers and frequency bands. The normality criterion was not passed for the set of values in band 31–40 Hz. For this reason, this band was not used in posterior analysis. For the ANOVA test, there were no conditional differences. There were independent differences for frequency bands and classifiers with a *p* < 0.01. To analyze the differences between methodologies, a *t*-test was applied to the data. Regarding the frequency bands: 8–40 Hz was significantly different from 8–14 Hz and 15–22 Hz, but not from 23–30 Hz. The 23–30 Hz was significantly different from the 8–14 Hz, but not from the rest. The average percentages for the bands were as follows: 8–14 Hz an average of 60.1%, 15–22 Hz an average of 60.7% and 23–30 Hz an average of 62.7% and 8–40 Hz an average of 64.9%.

Regarding the classifiers, there were significant differences between SVM and MDM and MDMfilter. There were no other differences. The average percentages for the classifiers were as follows: 60.6% for SVM, 63.1% for MDM, 62.6% for MDMfilter and 62.1% for LDA.

In addition, an analysis by accuracy filtering and randomness level allowed to detect the level above randomness for each configuration. This level was used to determine when the model performed acceptably in the classification of both classes. Table 3 shows the results according to the type of classifier, the type of filtering and the specific band for those cases in which the admission criterion was passed. The first value was the mean among subjects, the second was the number of valid subjects for that case and the third one was the deviation among the valid results. The mean of valid values per user averaging all the classifiers is higher for ASR in the band 8–14 Hz, 15–22 Hz and 23–30 Hz, while in $H^{\infty }$ the band 23–30 Hz and 8–40 Hz was higher. For the averages by bands of classifiers, the SVM obtains an average of valid values per user of 2.8 in the $H^{\infty }$, while in the ASR, a value of four. For the rest of the classifiers, averaging the valid values per user depending on the frequency filters was similar.

The standard deviation values were lower in the ASR, mainly because the values of the good subjects were lower. The band with the highest values per subject was 8–40 Hz in both filters. The classifier and band with the highest accuracy and value was 8–40 Hz in MDMfilter for the two types of artifact filters.

In Table 4, the selection of ASR and Hinfinity in the best band and classifier was compared by subject. In addition, an individual selection (personalized) of the best classifier and the best band per subject was added to the right side of the table. For the generic configuration, the average balance parameter and accuracy was 2.6 and 66.2% for $H^{\infty }$ and 5.5 and 65.1% for ASR, while the mean for personalized was 3.9 and 69.6% for $H^{\infty }$ and 4.4 and 68.2% for ASR. Among the solutions for generic, $H^{\infty }$ had the six best solutions for subjects in accuracy while ASR only had two. Among the solutions for personalized, $H^{\infty }$ had the five best solutions for subjects in accuracy, while ASR only had two. For $H^{\infty }$, the most selected classifier in the personalized configuration was MDMfilter three times. The 8–40 Hz band was the most chosen four times. For ASR, the most chosen classifier was MDMfilter three times. The most chosen band was 23–30 Hz three times.

**Figure 4:**
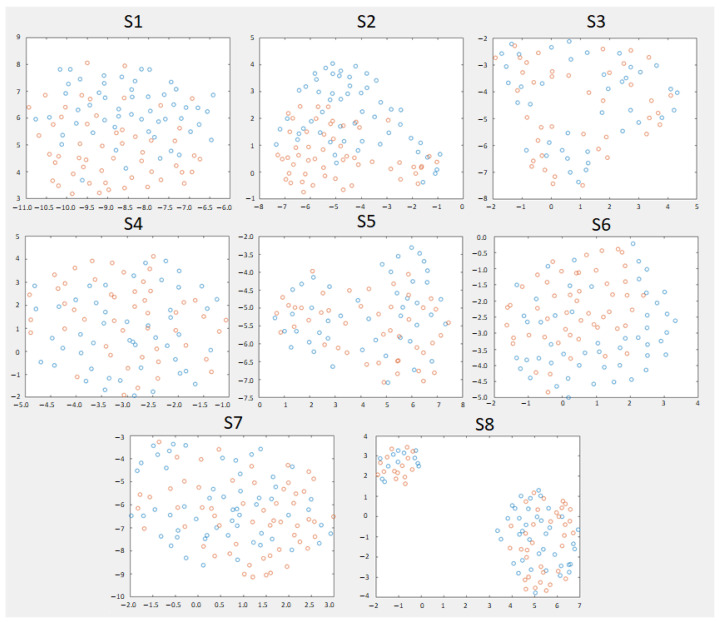
The 2D dimensionality Riemannian reduction in the Riemannian tangent space of eight trials chosen to make the model of each subject. The bands chosen for each model are those of the personalized configuration $H^{\infty }$. In blue, the epochs of monotonous walk and in orange the epochs of intention to change direction.

For each subject, the features of the tangent space of the covariance matrix were displayed. In Figure 4, it is shown the personalized configuration for $H^{\infty }$ that obtained the best results of Table 4. Based on this 2D representation made with UMAP [33], the subjects with the clearest visual separation were S1, S2, and S6. S1 and S2 were the ones with the most separated clusters. S2 had two extreme groups of both classes well separated up and down and mixed a little bit at the middle. For S8, there were two clusters with data from both classes, which may indicate a certain change in the non-stationary data.

These two configuration selection, generic and personalized, refer to the two temporary needs when generating a model. The next paragraphs will analyze different variants for the personalized configurations in the $H^{\infty }$ band. In Section 3.2.2, it will be evaluated if these configurations are relevant for real-time analysis.

##### Offline-Cross Validation Model Type

The results of the analysis for models using only right or only left turns were made from the best configurations (personalized) of the Table 4. In the Table 5, the comparison of the results for each of the models is shown for both the balance parameter and the *Acc* parameter.

The average of the highest classifier model was right and left with 69.6% and 3.9 balance. All configurations passed the randomness cutoff explained in Section 2.5.1. The models obtained worse averages in both accuracy and balance, and both had invalid results for some of the subjects. However, for some of the subjects, only the left or only the right model improved with respect to the left and right models. For subject S1, it improved slightly for the only right model, for S2 the left model improved the accuracy for left, for S5 the only right model improves, for S6 only the left model improved, also for S7.

##### Offline-Cross Validation Electrode Selection

**Figure 5:**
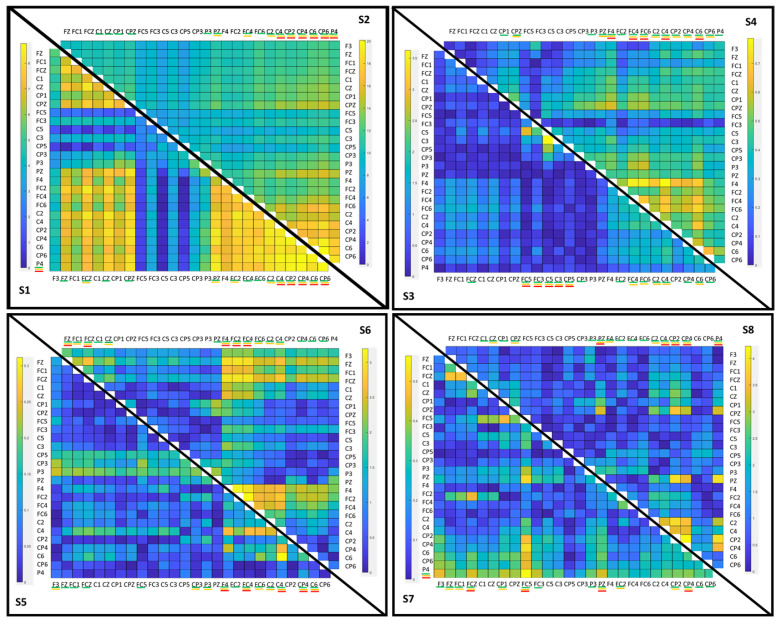
Color map of the upper diagonal of the matrix of differences between the mean of the matrices of the monotonous walk and change intention classes for each of the subjects in the best configurations in Table 4. The best electrodes for the selection of 15, 10 and 5 are indicated respectively underlined with colors: green, yellow and red.

Figure 5 shows, for each subject, the absolute value of the upper diagonal of the difference of covariance matrices between both classes. Depending on the subject, there was a greater or lesser difference in the scales of the values of the matrix. The subjects with the greatest difference in order were S2 and S1 and with less difference S8, S3 and S6. The rest had low values.

The most representative electrodes are those underlined in red (the 5 best) in Figure 5. The electrodes that appeared the most per subject (percentage values) were the frontal area (F4: 37.5%, FC4: 37.5%), motor area (C4: 62.5%, C6: 50%) and posterior parietal area on the right side (CP2: 37.5%, CP4: 62.5% and P4: 50%). Of this group of five electrodes, in subjects S1, S2, S4, and S5, there were only right-side electrodes. For S3, they were only on the left side. For S6 and S8, it was a combination of the central area and right side and for S7, it was a combination of the central area and both hemispheres.

The cross validation with the different numbers of electrodes is shown in the Table 6. In general, all the configurations were worse than having all the electrodes. Only one configuration improved on the personalized configuration of Table 4, for the S5 with 15 electrodes. There is no clear pattern of what is the best number of electrodes. The 5-electrode configuration was better than the rest of the electrode selection for two subjects, S2 and S4. The 10-electrode configuration was better for three subjects, S1, S3 and S7, as was the 15-electrode configuration for subjects S5, S6, and S8.

#### 3.2.2. Pseudo-Online Validation

Table 4 shows the offline-cross validation comparison of the results for the two conditions evaluated. The artifact filter chosen according to the conclusions of the previous section is the $H^{\infty }$. In Table 7, the pseudo-online results for both options are displayed.

It is important to take into account the ratio of FP/min and TP as the two most relevant measures to assess the BMI. However, the FPRatio and TPnoFP also give us important information. To evaluate the correct performance of the BMI, the higher the TP the better. With FP, the lower the better. For the FPRatio, the lower the better and for the TPnoFP, the higher the better.

The average of TP, FP/min, FPRatio and TPnoFP for the generic configuration is, respectively, 41.4%, 9.4, 80.1% and 2.3%, while in personalized, it is, respectively, 40.2%, 9.3, 80.1% and 4.0%.

Figure 3 shows the temporal distribution of the temporal analysis of the pseudo-online. In red, the FP with respect to the change in 0 s. In green, the TP that entered the line considered in purple. In blue, the furthest temporal repetition, in yellow, the average time that the repetitions last. According to Figure 3, S1 was the one with the highest signal evaluated and S3 the second. For subjects S1, S2, and S6, the FPs accumulated near the activation limit. This pattern can also be observed, although less evidently, with S8. S2 is the best subject, TP is moderate but FPs are very low and some of them fall near the change instant.

Regarding the FPRatio, the lowest was for S2 37.5%, followed by S4 with 78.6%, S6 with value of 81.3% and S1 with a percentage of 84.6%. According to the average FPRatio of 80.1% on average, although very variable, for 20% of the trials there was no FP, but not necessarily a TP either. S2 has 62.5% of trials without FP and for S4 and S5, although they do not have a good TP/FP/min ratio, there are 20% of trials in which there was no FP, which means that there may be FP concentrated in trials in which the subject was not sufficiently concentrated. Additionally, in these configurations, these three subjects are the only ones that have TPnoFP. S2 with 12.5%, as well as S6 and S1 with 7.7%.

It may be that the FPs are concentrated in trials that are bad, and in some there are none. Additionally, if there are clean trials of FP with a TP, it indicates that the BMI could work well and that its accuracy could depend on the concentration of the trial [23].

However, the classifier temporarily does not seem to discriminate the classes for S3, S5, S7 and S4. For S5, in addition, close to the turn, no detection appears.

### 3.3. Online EEG Validation

This section shows the test results in online test results of the subject S2. The subject made 28 turns, which were evaluated in a 1st phase of false positives and in a 2nd phase in which the ability of the interface to detect the turn was evaluated.

The model was created with 64 repetitions for S2, of which only one was automatically discarded with the IMU discard algorithm (later confirmed by the expert as the only wrong label). The rest was processed and the results for all bands and classifiers were processed in a cross validation as explained in Section 2.5.1. The best cross-validation result was in the 8–40 band with the SVM classifier. The result was *b* 3.6 and *Acc* 77.7% ± 12.4, AccMonotonousWalk, 80.3% ± 18.9 and AccChangeIntention 75% ± 14.4. The model was created in this band and with this classifier.

**Figure 6:**
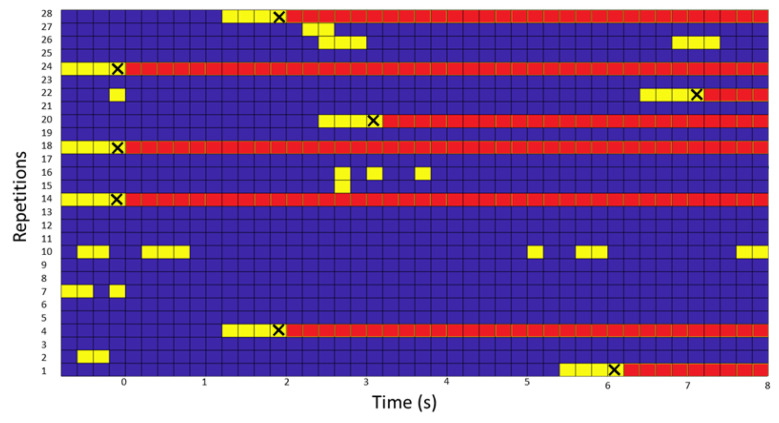
Figure with the FP of the subject who performed the test in real time. On the Y-axis, the number of repetitions performed and on the X-axis, the time elapsed since the start of the task that supports the sending of BMI commands. If the BMI detects the class intention to turn, the instant (frame) is displayed in yellow. If four consecutive FPs occurred, the person was forced to turn (instant of the verbal command marked with an X) and in the figure the remaining moments are plotted in red.

For the 1st phase in which the FP are evaluated, of 28 turns that were recorded, in 8 turns an FP was detected. Of all the recorded times, the interface failed 2.5 FP/min. According to Figure 6, the X-axis represents the elapsed time and the Y-axis the number of repetitions. In three of them, from the beginning, there was FP, while for another three, one was in the middle and about two were at the end. In another 8, there was some PF, but it did not exceed the mode of four consecutive. In the rest, 13 of them, which means 46.4% of the repetitions were without any type of FP.

Therefore, of the 28 tests showed on the Table 8, 71.4% of them went on to the 2nd phase. In this phase, three scenarios are possible, in which two of them are considered FP and the third is not. If the success occurred after the detection of the IMUs, the time of the column is considered negative and if it occurred before, if it was much earlier, it is also considered wrong. In this phase, it was TP 100% of the time and no FP was computed. The average times in which four consecutive classifications were produced and therefore a command came out on the screen, were 2.01 (the first command of the 4) to 0.21 (the last command of the 4 consecutive) before the turn was detected in the IMUs.

## 4. Discussion

The first objective of this work was to design a robust algorithm for offline detection of the real turning moment by means of IMUs. In addition to validate the performance of the algorithm, a discard algorithm that detects the correct repetitions and eliminates the invalid ones was designed. The discard method for labeling the IMUs was carried out by a visual inspection. The decision of the visual inspection and the algorithm was contrasted. Although the algorithm detected as invalid more repetitions than the visual inspection, which the algorithm corroborated, it only failed in one. Although in Section 2.5.1, it was only used as a reference, it was actively used for the online test for the direct creation of the model.

The second and the most important objective for this study is to detect the intention to turn in an online test from the EEG signal. Many configurations were tested to try to maximize the accuracy and performance of a BMI only commanded by the subject’s brain activity.

ASR and $H^{\infty }$ are two of the most popular algorithms in the literature, and in this work, it was evaluated how they can affect the creation of a model. The first ASR algorithm uses a shifting PCA window on covariance matrices to remove large amplitude artifacts potentially, while the second focuses on ocular artifacts and drift. For the extraction of features and the creation of the model, the covariance matrices and several Riemannian manifold classifiers were applied. This together with CSP with its variants are two of the most used algorithms in the state of the art. The CSPs were discarded due to the low number of repetitions of each of the classes and did not obtain good results in earlier experiments. The spatial/temporal features were prioritized before the frequency ones. According to our analysis, the features in frequencies obtained very variable results [12].

In addition, in this work, different frequency bands were evaluated to examine where the correlates of intention differ to a greater degree from the correlates of the monotonous walk. The filters used were by state variables to later be able to apply them in real time. In these frequency bands, the covariance matrix for both classes was extracted in 1.2 s windows and the patterns of both classes were classified with different Riemannian classifiers.

Several aspects of the statistical analysis stand out. First, the significance that exists between subjects is highlighted. There are bands significantly better than others; 8–40 Hz and 23–30 Hz seem to be the most significant. Furthermore, it seems that there are classifiers that under all conditions perform better than the rest, MDMfilter and MDM.

From this analysis, it is also concluded that there are no significant differences for $H^{\infty }$ or ASR. Both algorithms remove artifacts that could influence the turn decision (although the subject was trained not to do so). However, for the selection of a configuration, one must be chosen based on some criteria.

From the analysis of Table 3 it is concluded that the band and the classifier with the highest percentage and with the greatest number of valid configurations is the MDMfilter in the band 8–40 Hz in general. To choose which filter is selected in the Table 4, the mean of $H^{\infty }$ is higher both for the generic configuration and the lower balance, as well as for the personalized configuration.

The generic and personalized settings were tested in pseudo-online mode. The former takes less time than the latter to perform an online test. However, the personalized configuration has less FP and a higher percentage of TPnoFP. Therefore, for the test carried out in this study, this previous analysis will be carried out.

The results of the pseudo-online analysis were promising for three of the subjects. This is promising for online testing: respectively, the results of TP of 43.8%, FP/min 2.9 and TPnoFP of 12.5% for S2; S1 with TP 61.5%, FP/min 8.0 and TPnoFP 7.7%; and S6 with TP 43.8%, FP/min 7.5 and TPnoFP 12.5%. In addition to Figure 3, it is extracted that the FP/min accumulated near the validation point.

For the personalized configuration, several variations were also tested:

- First, it was tested whether it was easier to predict a model with only turn right or only turn left or both. The average of the results was better for both. Although right or left improved for some subjects, these settings are generally worse for all subjects.
- Second, a selection of electrodes was proposed. With this selection, although the results did not improve, the areas that most differentiate both classes were analyzed. The right hemisphere is the one that most differentiates this pattern. The posterior parietal cortex area is the most relevant, and this also agrees with what is expressed in other paradigms of intention [34].

The ability of S2 to modulate its brain activity allows for robust EEG classification. The online test obtains very good results, according to the FP/min metric used (once an FP was sent, the person turned and stopped). The designed experiment allows to accurately evaluate the conditions of the interface and can be easily extrapolated to the closed loop, as has already been done in similar experiments [12]. The FP/min obtained in this 2.5 test is one of the lowest results in the literature, also taking into account the features of this study; only information prior to the change is used, and only information from EEG brain activity is used [15,17]. The subjects of this study show more variability than in those mentioned studies, but this trend can be observed in many other works [35] of the bibliography. Comparing the online open-loop test with other works, two-state stop–start (no transition between two states with motion), in this work, information only before the real movement was used. Comparing with the jobs that do have a transition between two states with movement and online evaluation [17], apart from using only information from before, the IMUs were not used to lower FPs.

### Limitations and Future Work

Multiple factors may be conditioning our study and introducing undesired variability. Below are some of the most important factors to take into account for improvement and future proposals.

Regarding registration equipment, the signal was recorded wirelessly, rather than being wired, to offer mobility to the subject and avoid tracking problems. However, although electronic devices external to the registry were avoided, in the room, the wireless signal can be affected by electrical sources and radio signals.

In this study, a wide variety of algorithms were tested to try to maximize the performance of subjects, acting over the pre-processing, processing and classification methods. Regarding the filters used in pre-processing, many algorithms in the literature competed for artifact mitigation. Some of them used their own signal to discard noisy components, typically Laplacian [12], CAR [36] and MPCA [37]. These methods mix the variance between channels and impair the distinction of covariance matrices, which makes them incompatible with Riemannian tangent space algorithms. Other methods use a baseline to remove noisy components, such as ASR, or even another kind of biosignal to remove interference, e.g., $H^{\infty }$. These last two were tested in the paper.

Frequency filters were applied by state variable models. This choice was made to make the BMI compatible with previous BMIs [9] and work in real time. The final objective is to merge paradigms obtaining a total movement control by the BMI. Other methodologies, such as variational mode decomposition [37,38] and empirical wavelet transform [15,39], could be interesting techniques to analyze the rhythms in frequency–time and show highly accurate results [6]. However, its implementation in the current architecture would require further code optimization and computing resources to assure that each epoch iteration can be processed at a 0.2 s pace, which is the needed time for a proper average.

Regarding processing and classification, the classifiers tested were based on Riemmanian geometry, which is a current topic of interest in the field [40]. In previous phases of the study, other algorithms, such as neural networks, that require large amounts of data were discarded, as this paradigm has a limited amount of valid signal samples that can represent the neural state of intention to turn.

It is important to notice, that after testing many configurations, subject dependency was high. This is a general problem of EEG analysis [41], and hard to overcome. Subjects that did not obtain good results had really difficulties to improve. Thus, other alternatives should be explored to try to improve and standardize the variability of the results. Some of the aspects that should be considered are as follows.

Although in other paradigms, such as MI, it has been reported that improvement can occur during the week based on a plasticity phenomenon, the improvement through intention paradigms is not clear. Nevertheless, Gangadhar et al. [42] reported an improvement after multiple sessions.

Although the instructions on mental states were clear, some of the subjects performed the mental process in a different way. Protocols should be improved, making special emphasis in the messages to the subjects, guiding them in a more strict way in the explanation of the mental processes. FPs could be reduced, standardizing the mental states that can occur during the monotonous walk phase. Different cognitive states could be tested in addition to a totally spontaneous turn, being aware of making the decision. Using a continuous mental process of preparation for the turn would avoid relying on a spontaneous intention event. This could be a more robust and easier way to detect the turn, because the window in which this cognitive phenomenon occurs is larger that the one when it is totally spontaneous [43]. These factors could produce a greater improvement than the improvement that sophisticated processing or classification algorithms could produce [44].

The online validation chosen has a slightly different conditioning factor than the pseudo-online analysis might have. This may also explain why a smaller mode (four) S2 real-time experiment fits better than the pseudo-online analysis (five). However, the online validation, as discussed above, allows a very simple validation that is easy to extrapolate to the use of assistive devices [12].

The objective is to apply this biomarker on patients with some degree of disability, whose training can be difficult to do for enough trials. The logical step to apply this to any type of patient is to try to make the EEG model independent of the subject. Nevertheless, transfer learning has limitations in the application of EEG [45], and especially for this paradigm, as there are many sources of variability (different turn intention pattern and label trend). Other alternatives, such as the generation of repetitions from GANS [46], could be more appropriate to subsequently apply neural network methodologies. The control would be performed with an assistive device, and the EEG model would be retrained in real time. It is proposed to create a generic model and to set up a real-time adaptation prototype.

As this experiment has a dual application in both the rehabilitation and care domains, an attempt should be made to improve the robustness of the system. By modeling the EEG as two classes, other point mental states (or noise) that were not in the training may be mistaken our classification. This can be seen in Figure 4. Although for some subjects, the classes were very mixed, for those who obtained better results, see results in bold Table 7, the UMAP algorithm represented the classes separately, except for some samples in the intermediate state. Therefore, having several uncertainty states (that favor the non-activation of the device) enveloping our data may be an effective way to achieve greater robustness for real-time use, outside of a controlled experimental setting.

## 5. Conclusions

In this work, the possibility of online modeling the EEG signal for the intention to turn was explored. For this, an algorithm for detecting the angle of rotation was designed through IMUs inertial sensors. In addition, a whole protocol for the processing of the EEG through several analysis of the most referenced algorithms in the bibliography was proposed. A BMI was designed that detects the intention to turn only through EEG in real time with a low FP/min rate and a high success rate. In future studies, the issue of standardizing the results of the subjects and proposing models applicable to patients should be addressed.

## Figures and Tables

**Table 1:** Table with the discarded repetitions for each subject, according to the specified criteria: if there were failures in the protocol, if the algorithm mislabeled any repetition, or if in the labeled repetition there was not enough monotonous walk time.

Subjects	Discarded Repetitions			Valid Repetitions
	Protocol Discarded	IMUs Algorithms Discarded	Time Discarded	
S1	-	6	-	74/80
S2	1	3	1	75/80
S3	-	16	-	72/88
S4	-	10	-	70/80
S5	2	18	-	68/88
S6	-	5	-	75/80
S7	-	5	1	74/80
S8	-	9	-	71/80

**Table 2:** Table with the comparison between the discard algorithm and the visual inspection. In the first row, number of repetitions in which the criterion coincided between the algorithm and the visual inspection; in the second row number of repetitions in which the criterion was only applied by the algorithm; in the second row, number of repetitions in which the criterion was only applied in the visual inspection.

IMUs Algorithm Labeled Methodology Discard	S1	S2	S3	S4	S5	S6	S7	S8
Both	5	3	16	10	16	5	5	9
Only algorithm	5	2	0	5	8	3	3	6
Only visual inspection	1	0	0	0	0	0	0	0

**Table 3:** Table with the average number of valid solutions per subject, depending on the classifier, artifact filter and frequency band. Filter settings with highest accuracy and value are highlighted in bold.

Classifiers	Filters	8–14 Hz	15–22 Hz	23–30 Hz	31–40 Hz	8–40 Hz
MDM	$H^{\infty }$	65.8 (4) ± 3.9	69.0 (3) ± 9.8	67.5 (5) ± 7.9	75.9 (2) ± 13.9	72.0 (5) ± 11.7
	ASR	70.3 (4) ± 4.9	68.8 (4) ± 1.5	65.1 (4) ± 7.4	66.7 (3) ± 8.0	67.7 (6) ± 6.4
MDMfilter	$H^{\infty }$	65.6 (5) ± 4.7	71.9 (2) ± 6.9	68.1 (5) ± 8.2	75.4 (2) ± 18.3	**70.0 (6) ± 9.9**
	ASR	66.9 (5) ± 5.3	69.4 (4) ± 3.7	70.9 (3) ± 3.3	68.2 (3) ± 9.8	**68.4 (6) ± 7.6**
lda	$H^{\infty }$	67.5 (5) ± 4.2	67.4 (4) ± 9.1	66.7 (6) ± 7.3	75.4 (2) ± 14.5	67.9 (5) ± 11.1
	ASR	67.8 (4) ± 5.9	66.9 (5) ± 5.3	65.5 (4) ± 3.4	68.5 (3) ± 10.7	72.3 (4) ± 5.9
SVM	$H^{\infty }$	67.0 (2) ± 0.0	68.2 (3) ± 12.2	71.3 (3) ± 8.1	87.5 (1) ± 0.0	66.8 (5) ± 12.7
	ASR	63.3 (5) ± 3.9	67.9 (4) ± 5.3	66.9 (4) ± 4.4	66.9 (3) ± 9.4	70.5 (4) ± 7.4

**Table 4:** Table with the configuration per subject, on the left the configuration selected from the previous table and on the right the best configuration per subject, specifying the filter and band in which it was produced. The solution is accompanied by an asterisk, if it did not pass the accuracy filter greater than randomness or balance.

Subjects	Filters	Generic Configuration				Personalized Configurations			
		*b*	*Acc*	Classifier	Band (Hz)	*b*	*Acc*	Classifier	Band (Hz)
S1	$H^{\infty }$	6.0	75.2	MDMfilter	8_40	6.1	75.4	MDMfilter	8_14
	ASR	6.4	74.1	MDMfilter	8_40	7.5	74.8	MDM	8_14
S2	$H^{\infty }$	2.9	88.4	MDMfilter	8_40	0.0	89.3	SVM	8_40
	ASR	5.3	79.5	MDMfilter	8_40	0.0	80.4	lda	31_40
S3	$H^{\infty }$	3.3	64.6	MDMfilter	8_40	3.3	64.6	MDMfilter	8_40
	ASR	9.6	59.4	MDMfilter	8_40	3.3	60.4	SVM	31_40
S4	$H^{\infty }$	1.6	60.0	MDMfilter	8_40	4.6	60.7	lda	23_30
	ASR	0.0	57.1 *	MDMfilter	8_40	1.4	63.4	SVM	23_30
S5	$H^{\infty }$	4.0	50 *	MDMfilter	8_40	6.7	60.0	MDMfilter	15_22
	ASR	5.6	53.1 *	MDMfilter	8_40	7.4	60.0	MDMfilter	8_14
S6	$H^{\infty }$	0.0	66.1	MDMfilter	8_40	6.3	69.6	MDM	8_40
	ASR	6.9	68.8	MDMfilter	8_40	6.9	68.8	MDMfilter	8_40
S7	$H^{\infty }$	2.8	57.8 *	MDMfilter	8_40	1.2	67.8	SVM	23_30
	ASR	1.3	61.7	MDMfilter	8_40	4.9	67.2	MDMfilter	23_30
S8	$H^{\infty }$	0.0	67.9	MDMfilter	8_40	3.1	69.6	MDM	8_40
	ASR	8.5	67.0	MDMfilter	8_40	3.4	70.9	MDM	23_30

**Table 5:** Table with each type of model: for only right turn, only left turn and Both. Asterisk indicates that it did not pass the criterion of valid result. In bold, the best configurations.

	Right		Left		Right and Left	
	*b*	*Acc*	*b*	*Acc*	*b*	*Acc*
S1	**9.4**	**76.6**	10.0	70.8	6,1	75.4
S2	0.0	83.3	**2.2**	**93.8**	0.0	89.3
S3	25.0 *	52.1 *	50.0 *	65.6	**3.3**	**64.6**
S4	4.2	52.1 *	8.3	54.2 *	**4.6**	**60.7**
S5	7.7	75.0	7.7	43.8 *	**6.7**	**60.0**
S6	0.0	66.7	**10.0**	**81.3**	6.3	69.6
S7	15.6 *	56.3 *	**9.9**	**73.4**	1.2	67.8
S8	5.6	59.4 *	4.5	65.6	**3.1**	**69.6**

**Table 6:** The best electrodes for the selection of 15, 10 and 5 are indicated respectively underlined with colors: green, blue and red. The best results of the three electrode configurations have been highlighted in bold.

Subjects	15		10		5	
	*b*	*Acc*	*b*	*Acc*	*b*	*Acc*
S1	0.0	69.6	**1.3**	**70.5**	3.9	70.5
S2	0.0	80.4	1.1	81.2	**8.0**	**82.1**
S3	5.8	51.0	**1.9**	**53.1**	14.6	57.3
S4	3.3	51.8	0.0	50.0	**1.7**	**52.7**
S5	**4.8**	**61.5**	6.5	60.4	7.1	54.2
S6	**1.4**	**65.2**	2.8	62.5	1.6	58.0
S7	1.4	57.0	**2.9**	**56.2**	9.2	53.9
S8	**4.7**	**59.8**	3.1	55.4	6.3	53.6

**Table 7:** Table with the results of the pseudo-online analysis: in the first row for the generic configuration and in the second row the customized one for each of the metrics. The configurations of the three users with repetitions with TP and without FP have ben highlighted in bold.

Subject	Option Selected	TP (%)	FP/min	FP Ratio (%)	TPnoFP
S1	Generic	69.2	8.0	92.3	0.0
	Personalized	**61.5**	**8.0**	**84.6**	**7.7**
S2	Generic	**43.8**	**3.6**	**50.0**	**6.3**
	Personalized	**43.8**	**2.9**	**37.5**	**12.5**
S3	Generic	56.3	17.2	100.0	0.0
	Personalized	56.3	17.2	100.0	0.0
S4	Generic	14.3	7.8	64.3	0.0
	Personalized	35.7	9.6	78.6	0.0
S5	Generic	20.0	10.9	93.3	0.0
	Personalized	13.3	7.8	80.0	0.0
S6	Generic	**43.8**	**8.6**	**75.0**	**12.5**
	Personalized	**43.8**	**7.5**	**81.3**	**12.5**
S7	Generic	20.0	7.7	73.3	0.0
	Personalized	26.7	12.3	93.3	0.0
S8	Generic	64.3	11.5	92.9	0.0
	Personalized	40.7	9.2	85.7	0.0

**Table 8:** Table with the results of the 1st phase and 2nd phase of the online test. Results of each of the repetitions. If in the 1st phase, there was a PF, it is indicated with a 1 in the first phase and the time it took to happen. If in the 2nd phase there was a TP, it is indicated with a 1 and the interval in which it occurred, with a + sign if it was before the real turn or with a - sign if it was after the real turn. If a command occurred in the 2nd phase outside the valid metrics, FP was counted with a 1, otherwise a 0.

Repetition	1st Stage		2nd Stage			
	FP	FP Walk Time (s)	FP	TP	Window Interval (s)	
1	1	7.00	-	-	-	-
2	0	-	0	1	2.02	0.22
3	0	-	0	1	2.04	0.24
4	1	2.80	-	-	-	-
5	0	-	0	1	1.86	0.06
6	0	-	0	1	2.36	0.56
7	0	-	0	1	1.86	0.06
8	0	-	0	1	1.96	0.16
9	0	-	0	1	1.91	0.11
10	0	-	0	1	2.11	0.31
11	0	-	0	1	1.80	0.00
12	0	-	0	1	1.92	0.12
13	0	-	0	1	1.96	0.16
14	1	0.80	-	-	-	-
15	0	-	0	1	2.15	0.35
16	0	-	0	1	2.09	0.29
17	0	-	0	1	2.11	0.31
18	1	0.80	-	-	-	-
19	0	-	0	1	1.92	0.12
20	1	0.76	-	-	-	-
21	0	-	0	1	2.07	0.27
22	1	8.00	-	-	-	-
23	0	-	0	1	2.03	0.23
24	1	0.80	-	-	-	-
25	0	-	0	1	1.97	0.17
26	0	-	0	1	1.96	0.16
27	0	-	0	1	2.03	0.23
28	1	2.80	-	-	-	-

## Data Availability

Not applicable.
